# Secure energy outage analysis of UAV swarm-based network

**DOI:** 10.1371/journal.pone.0325785

**Published:** 2025-06-13

**Authors:** Tien Hoa Nguyen, Hoang Vu Tran

**Affiliations:** 1 School of Electrical and Electronic Engineering, Hanoi University of Science and Technology, Hanoi, Vietnam; 2 The University of Danang - University of Technology and Education, Danang, Vietnam; Northwestern Polytechnical University, CHINA

## Abstract

In the pursuit of enhancing energy-efficient and secure communications, this study explores secure energy outage probability (SEP) within unmanned aerial vehicle (UAV) swarm-based energy harvesting networks. UAVs, often deployed as relay nodes to facilitate communication between a source and a destination, face significant security and energy efficiency challenges, particularly when relying on battery-free configurations. These UAVs must simultaneously harvest energy from ambient sources and thwart eavesdropping attempts from external interceptors, demanding secure solutions for efficient operation. To address these challenges, we propose two energy-efficient secure UAV selection methods aimed at optimizing the communication network’s physical layer security. The first method prioritizes maximizing the communication link quality between the source and the selected UAV, while the second method focuses on minimizing the link quality between the UAV and the eavesdropper. Through these strategies, the study targets improved SEP performance without compromising energy efficiency, ensuring sustainable network operation. The study evaluates the effectiveness of these methods through rigorous mathematical frameworks in both approximate and asymptotic forms. Through numerical analyses, our proposed methods demonstrate notable improvements in SEP performance. Specifically, we observe that SEP increases significantly when the transmit signal-to-noise ratio (SNR) is varied from low to medium values; however, the benefits taper off at higher SNR ranges, indicating a performance plateau. Moreover, introducing a larger number of UAVs in the swarm enhances SEP, showcasing the scalability advantage of the proposed methods. Further insights reveal that configuring a higher power-splitting coefficient enhances SEP at lower SNR values, although it yields diminishing returns at higher SNR levels. Additionally, our findings highlight the influence of UAV placement, with closer proximity to the destination improving SEP performance. However, there exists an optimal flying altitude for UAVs that minimizes SEP, offering a balance between security enhancement and energy efficiency. These results underscore the superiority of the proposed methods in addressing the dual challenges of physical layer security and energy conservation.

## 1 Introduction

### 1.1 Background and motivation

In recent years, autonomous aerial vehicles (UAVs) have revolutionized radio access networks, serving as a beacon for the future of coordinated multipoint and cloud-based communication systems [1,2]. These multi-role aircraft play a pivotal role in swiftly establishing temporary connections for enhanced wireless communications, surveillance missions, disaster relief efforts, and delivery operations [3,4]. UAV-assisted communication has garnered immense interest due to its potential to significantly boost coverage, reliability, and throughput for ground users. For instance, the ability to deploy multiple UAVs to serve ground users has been demonstrated from various perspectives [5] such as optimizing the interplay of multiuser communication scheduling, user association, UAV trajectory, and power control to ensure the highest minimum throughput. Additionally, studies on comprehensive channel measurements and modelling for UAV communications [6] have been popularity radiated from not only air-to-ground and ground-to-ground but also air-to-air. Besides, protocols like collaborative UAV sense-and-send have also been launched in [7] to enable diverse UAV-to-X communications, tackling subchannel allocation and UAV speed optimization to maximize the uplink sum rate. Moreover, the performance comparison of spectrum-sharing mechanisms in UAV-to-UAV transmission-reception pairs has been scrutinized [8]. Another research in [9] studied how to reduce the number of UAVs needed while enhancing coverage rates by optimizing UAV trajectory, user clustering, and frequency band allocation. Meanwhile, initiatives in [10] proposed a three-hop UAV-aided non-orthogonal multiple access (NOMA) network framework to support massive connections, heightened spectral efficiency, and flexibility. Investigations in [11] have extended to millimetre Wave (mmWave) and sub-6 GHz frequencies for robust wireless fronthaul connections between UAVs and terrestrial networks, considering various blockage environments and UAV altitudes.

However, despite their impressive capabilities, UAV-enabled communications encounter several challenges, such as limited onboard energy, mobility constraints, channel impairments, and interference management [12]. Among these, the limitation in onboard energy is particularly critical as it directly influences the sustainability of UAV operations [13]. To address this issue, UAVs can replenish their energy through ambient energy harvesting (EH) sources like wind and solar, or via radio frequency wireless charging, which offers greater flexibility and allows energy harvesting from available radio resources [14]. Radio wireless charging technologies can be categorized into simultaneous wireless information and power transfer (SWIPT) and wireless power communication networks [15]. SWIPT, which employs time-switching and power-splitting (PS) mechanisms to harvest energy from information signals transmitted by ground base stations, provides more seamless and convenient communications over wireless power communication networks, which separate the processes of wireless energy transfer and information transmission.

The scientific community has shown a keen interest in the potential of SWIPT for UAV-enabled communications. Research in [16] indicated that networks bolstered by multiple UAV relays can significantly improve IoT systems by mitigating large-scale fading between the source and sink. This improvement is achieved through green cooperative communications employing TS and PS strategies to enhance signal reception at the terminal node. Additionally, studies in [17] delved into energy-constrained UAV-enabled secure communication systems featuring multiple eavesdroppers, optimizing UAV positioning, artificial noise transmit power, and PS and TS ratios to elevate secrecy rates. Further explorations focused on refining PS and time allocation to minimize outage probability in data transmission [18], as well as proposing integrated content caching and EH schemes [19] to strengthen UAV communications within IoT networks. Recently, UAV-enabled SWIPT communications has received considerable interest in developing new communication paradigms. For example, the authors in [20] studied maximizing the energy efficiency of a cooperative terahertz network where the miniature UAV employs SWIPT-based PS to receive information and power simultaneously. To do so, the authors proposed a quadratic transform approach to jointly optimizing the UAV trajectory, the PS ratio, and NOMA power allocation for multi-user communication, and it is demonstrated that the proposed method can achieve higher energy efficiency compared to the baselines. In [21], a multi-antenna AIRS (UAV integrated intelligent reflective surface)-assisted SWIPT system with UAV jitter was put into use, in which two reflective surfaces were deployed on two UAVs to reflect the signals transmitted from the base station to the information user and the energy user. To support more device connections and provide the necessary power to sensors at the cell edge in the agricultural Internet of Things, the authors in [22] introduced an integration technique that seamlessly combines the benefits of SWIPT, single-input multiple-output, and NOMA. In addition to this, they also improved the outage performance at cell-edge sensors by proposing a transmit antenna selection strategy for the base station. In [23], the authors studied how to simultaneously optimize the UAV’s trajectory, transmission power, and PS or time-switching policy to maximize the total logarithmic average throughput of the ground nodes while ensuring their average harvested energy demand under both linear and nonlinear models. In [24], the authors proposed a novel connectivity preserved multi-agent deep reinforcement learning algorithm to solve the trajectory planning problem in collaborative UAV in providing SWIPT services for ground users. In [25], the authors outlined an active reconfigurable intelligent surface-aided UAV-enabled SWIPT to partially compensate for multiplicative fading, typically encountering performance reduction in conventional passive architecture. In [26], the authors investigated maximizing the minimum information receivers’ rate in UAV-integrated reconfigurable intelligent surface with SWIPT services by jointly optimizing the UAV trajectory, precoding matrix, and reflective phase shifter. In [27], the authors highlighted the advantages of integrating covert transmission with joint security design to enhance UAV network secrecy, driving efforts to improve both energy efficiency and security in UAV swarm-enabled EH networks. For a comprehensive review on UAV-based EH mechanism, readers might refer to [28] and find insightful discussion therein.

On another front, ensuring the security of wireless communication systems remains a critical challenge due to the openness of wireless broadcasting and the mobility of UAVs [2]. Physical-layer (PHY) security has emerged as an effective and computationally efficient method to secure UAV communications. The principle of this technique is to use the randomness of the wireless channel to prevent information from being eavesdropped on [29]. Since there is no need for key distribution, which greatly saves the system cost, the encrypted transmission can achieve better service quality. As a result, the exploration of PHY security in UAV networks has emerged as a significant research focus in recent years [30]. Numerous scientists are investigating communication secrecy and privacy protection, recognizing PHY security transmission as a promising solution for safeguarding wireless transmission secrecy in the future [31]. This approach has garnered substantial acceptance within the scientific community [32].

For instance, the work in [33] investigates the resource allocation problem for a UAV-assisted secure SWIPT system. Additionally, the study in [34] examined a four-node setup with UAV-enabled relaying, where eavesdroppers with partially known locations pose a security threat, and explores the power allocation between the source and relay. Among various PHY-security strategies, cooperative jamming stands out as a viable technique to counter eavesdropping by transmitting jamming signals that degrade the quality of the wiretap channel. For example, in [35], the mobility of a UAV is leveraged to improve the achievable secrecy rate through trajectory design and power control optimization, demonstrating better performance than static jammer due to the ability to jam opportunistically at closer distances to the eavesdropper. Furthermore, the authors in [36] addressed the problem of maximizing the minimum secrecy rate in UAV communications by incorporating jammer, through joint optimization of the UAVs’ trajectory and transmit power. In [37], the author focused on employing a UAV as a friendly jammer to secure communications against unknown eavesdroppers, analyzing the impact of UAV-jammer displacement and power control on reliability and security.

Nonetheless, most research on UAV-enabled SWIPT communication in references [16–19] generally assumes that UAVs have sufficient energy for their operations and that all harvested energy from the EH process is dedicated solely to communication. This assumption is impractical when energy must be shared between communication and fundamental UAV operations, leading to an energy preservation challenge for battery-powered UAVs. To the best of the authors’ knowledge, only studies [38] and [39] addressed this issue. Specifically, the authors in [38] proposed to reduce energy outage events in simple cooperative UAV EH networks by using a UAV relaying selection scheme while the authors in [39] considered energy reduction by using a multiple antenna selection scheme. Meanwhile, no existing works have studied the energy outage probability of UAV systems while simultaneously considering security constraints.

### 1.2 Novelty and contributions

As seen from the above summary, studying the energy outage probability (SEP) performance of UAV swarm-aided energy harvesting networks is highly interesting and promising. In this paper, we consider two UAV selection protocols in which the best UAV is selected in the way of either the maximal link from the source to itself or the minimal link from itself to the eavesdropper. While Table 1 shows the comparison between our work and related papers, Table 2 shows the key notations used in this paper. The present work addresses this gap in the literature, with the following main contributions:

**Table 1:** The comparison of our work and the previous works.

Ref./Prop.	EH	UAV swarm	OP	SEP	Selection Scheme
[38]	Solar EH	Yes	Yes	No	UAV
[39]	SWIPT-PS	No	Yes	No	Antenna
Our study	SWIPT-PS	Yes	No	Yes	UAV (Two schemes)

**Table 2:** Main parameter notation.

Symbol	Notation	Symbol	Notation
$x_{U}$	Signal of ground user	$p_{S}$	Transmit power at Source
$m_{AB}$	Shape parameter of link from A to B	*K*	Number of UAV swarms
$\Omega_{AB}$	Scale parameter of link from A to B	ϵ	PS coefficient
$h_{AB}$	Channel gain from A to B	$d_{AB}$	Distance from A to B
$F_{AB}(x)$	Cumulative distribution function (CDF)	𝔼{•}	Expectation operator
$f_{AB}(x)$	Probability density function (PDF)	Pr{•}	Probability operator
$p_{R_{k}}$	Transmit power of UAV	$n_{AB}$	AWGN at node B
$P_{R_{k}}^{EH}$	Harvested transmit power	$\gamma_{AB}$	SNR from A to B
C(•,•)	Secure Shannon rate	T(•,•)	The transmission time
$P_{hover}$	Hovering power consumption	$E_{stor}$	The stored energy budget
$E_{marg}$	Marginal energy budge	*N*	Secure message size
*W*	Bandwidth	$\overset{―}{\gamma }$	Average transmit SNR
${𝒦}_{•}(•)$	Bessel function of the second kind	SEP	SEP metric

1. A first investigation on the secure energy perseveration of UAV swarm network under Nakagami-*m* fading channels, where we propose two UAV swarm-secured energy harvesting selection schemes that include the maximal link of source-UAV communication and the minimal link of UAV-eavesdropper communication.
2. To assess the SEP performance of two proposed UAV selection schemes, we derive the analytical approximation expression. Notably, by quantifying the high SNR regime, we carry out the asymptotic SEP, which reveals useful insights into the performance limit of the SEP.
3. We validate the correctness of the developed mathematical SEP frameworks by conducting several numerical evaluation results using the Monte-Carlo simulation method under various impacts of key parameters, including the transmit SNR, the number of UAV swarms, the PS setting, the use of UAV rotor design, and UAV trajectory.

### 1.3 Structure of the paper

Following the introduction in Sect 1, we present the system model of UAV swarm-aided energy harvesting networks in Sect 2, including the network operation, the channel modelling, and the proposed UAV selection schemes. In Sect 3, we provide the analytical expressions for the SEP expressions for two proposed UAV selection schemes and both are quantified under both approximation and asymptotic manners. Next, Sect 4 present numerical and simulation outcomes to validate the developed mathematical frameworks before concluding the paper with a summary of key findings and our contributions.

## 2 System model description

**Fig 1:**
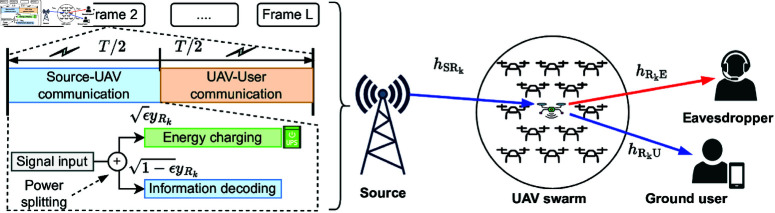
Illustration of UAV swarm-based energy harvesting networks.

As shown in Fig 1, we consider UAV swarm-based energy harvesting networks including a source S, *K* aerial UAVs (denoted by R), a ground user U, and an external eavesdropper (denoted by E). All nodes are assumed to be single antenna devices and there is no direct link between the source and ground user/eavesdropper due to obstacles. In the considered network, UAVs hover within a specific service area and act as decode-and-forward relay nodes for remedial communication between source and user in emergencies or supporting broadband service for temporary events [39]. However, aerial UAVs have limited storage capacity on the one hand, and the broadcast communication between the UAV and the ground user, on the other hand, is wiretapped by an eavesdropper.

To deal with such challenges, we consider UAVs adopting a SWIPT-based PS mechanism to recharge their energy budget, and among them, only one best UAV is selected to proceed with the cooperative communication. Details of the whole energy harvesting and communication process are described in the following subsection.

### 2.1 Channel modeling

In this work, we consider the channels from the source to the *k*-th UAV $h_{SR_{k}}$, from the *k*-th UAV to the user $h_{R_{k}U}$, and from the *k*-th UAV to the user $h_{R_{k}E}$ to be undergoing the Nakagami-*m* fading model, for its capability to effectively account for multipath fading, shadowing, and scattering effects resulting from obstacles, buildings, terrain, or weather conditions, while also reflecting various realistic channel types. Accordingly, the CDF and PDF of links from the transmitter $A\in \{S,R_{k}\}$ to the receiver $B\in \{R_{k},U,E\}$ can be modeled as

$$F_{|h_{AB}{|}^{2}}(x)=1-\exp\left(-\frac{m_{AB}}{\Omega_{AB}}x\right)\sum_{m=0}^{m_{AB}-1}\frac{1}{m!}{\left(\frac{m_{AB}}{\Omega_{AB}}x\right)}^{m},$$ (1)

$$f_{|h_{AB}{|}^{2}}(x)=\frac{x^{m_{AB}-1}}{\Gamma (m_{AB})}{\left(\frac{m_{AB}}{\Omega_{AB}}\right)}^{m_{AB}}\exp\left(-\frac{m_{AB}}{\Omega_{AB}}x\right),$$ (2)

where $m_{AB}$ and $\Omega_{AB}$ are the shape and scale parameters, respectively. Taking into account both small and large scale fadings, $\Omega_{AB}$ can be modeled as

$$\Omega_{AB}=G_{AB}D_{AB},$$ (3)

where $G_{AB}$ stands for the transceiver gain and $D_{AB}$ represents the path loss coefficient, with

$$D_{AB}=d_{AB}^{-\rho }(\lambda /[4\pi ]{)}^{2},$$ (4)

where λ implies the wavelength radiated RF signal, $d_{AB}$ is the average physical distance, and ρ∈[2,6] is the path-loss exponent.

### 2.2 Energy charging and data communication

#### 2.2.1 Source-UAV communication.

During the first communication stage (source-UAV communication), the source transmits signal $x_{U}$, with $𝔼\{|x_{U}|\}=0$ and $𝔼\{|x_{U}{|}^{2}\}=1$, to the *k*-th UAV with the transmit power $p_{S}$ over channel $h_{SR_{k}}$ and the signal power received by the *k*-th UAV can be expressed as

$$y_{R_{k}}=\sqrt{p_{S}}x_{U}h_{SR_{k}}+n_{R_{k}},$$ (5)

where $n_{R_{k}}\sim CN(0,\sigma^{2})$ is the additive white Gaussian noise (AWGN) with zero means and variance $\sigma^{2}$.

Under the SWIPT-based PS mechanism in Fig 1, the UAV simultaneously processes energy harvesting and information decoding upon a received signal $y_{R_{k}}$ from the source. Specifically, the UAV splits it into two streams: one designated for energy harvesting and the other for information decoding. The PS mechanism manages this division, allowing the UAV to allocate the PS ratio ϵ to charge its energy storage system while using the remaining portion (1−ϵ) to decode the data. Accordingly, the usable electrical power harvested from RF signals $y_{R_{k}}$ with the energy conversion efficiency μ and the PS ratio ϵ can be deduced as

$$P_{R_{k}}^{EH}=\mu \epsilon p_{S}|h_{SR_{k}}{|}^{2}.$$ (6)

Meanwhile, the remaining signal used for information decoding can be given by

$$y_{R_{k}}^{ID}=\sqrt{(1-\epsilon )p_{S}}x_{U}h_{SR_{k}}+n_{R_{k}}.$$ (7)

Thus, the output signal-to-noise ratio (SNR) at the *k*-th UAV can be expressed as

$$\gamma_{SR_{k}}=(1-\epsilon )\overset{―}{\gamma }|h_{SR_{k}}{|}^{2},$$ (8)

where $\overset{―}{\gamma }≜p_{S}/\sigma^{2}$ is the average transmit SNR.

#### 2.2.2 UAV-user communication.

After decoding $x_{U}$ during the first phase, the *k*-th UAV then encodes and transmits this information to ground users with the transmit power $p_{R_{k}}\leq P_{R_{k}}^{EH}$. The signals from the *k*-th UAV to the ground user and eavesdropper can be respectively expressed as

$$y_{U}=\sqrt{p_{R_{k}}}x_{U}h_{R_{k}U}+n_{U},\;y_{E}=\sqrt{p_{R_{k}}}x_{U}h_{R_{k}E}+n_{E},$$ (9)

where $n_{U}\sim CN(0,\sigma^{2})$ and $n_{E}\sim CN(0,\sigma^{2})$ are the AWGN components. The output SNRs at the ground user and eavesdropper can be respectively formulated as

$$\gamma_{R_{k}U}=\frac{p_{R_{k}}}{\sigma^{2}}|h_{R_{k}U}{|}^{2},\;\gamma_{R_{k}E}=\frac{p_{R_{k}}}{\sigma^{2}}|h_{R_{k}E}{|}^{2}.$$ (10)

### 2.3 Secure Energy Outage Probability (SEP) formulation

From (8) and (10), the secure Shannon rate in bit per second per hertz (bit/s/Hz) between the main and eavesdropping channels can be evaluated as


$$C(X_{k},Y_{k})={\left[\frac{1}{2}\log_{2}\left(1+X_{k}\right)-\frac{1}{2}\log_{2}\left(1+Y_{k}\right)\right]}^{+}$$

$$={\left[\frac{1}{2}\log_{2}\left(\frac{1+X_{k}}{1+Y_{k}}\right)\right]}^{+},$$ (11)

where $X_{k}≜\min\{\gamma_{SR_{k}},\gamma_{R_{k}U}\}$, $Y_{k}≜\gamma_{R_{k}E}$, and $\left[x{]}^{+}≜\max\{x,0\right\}$.

Suppose that the source transmits a message with *N* secured bits over the bandwidth *W* of access links in Hz, the transmission time for communication in second (s) can be derived as

$$T(X_{k},Y_{k})=\frac{N}{WC(X_{k},Y_{k})}.$$ (12)

To assist the cooperative communication effectively, the UAVs need to maintain a certain energy level for hovering in the sky. Considering its total weight *w* and impacts of gravity force, involving factors: the standard acceleration of gravity with $g=9.8\;m/s^{2}$, the typical density of air or atmospheric density $\alpha =1.225\;kg/m^{3}$, the area of the rotor disk β=0.2 $m^{2}$, and the number of rotors δ, the power consumed by the UAV can be modelled as in [38] as

$$P_{hover}=\sqrt{\left(wg{)}^{3}/[2\alpha \beta \delta \right]}.$$ (13)

Following that, the resource energy policy can be deduced from the harvested power $P_{R_{k}}^{EH}$ and hovering power consumption $P_{hover}$ can be established as

$$(P_{hover}+p_{R_{k}}-P_{R_{k}}^{EH})T(X_{k},Y_{k})\leq E_{stor}+E_{marg},$$ (14)

where $E_{stor}$ is the stored energy budget used for cooperative communication and $E_{marg}$ is the marginal energy budget used to maintain the basic operations of the UAV without interruption. Based on this policy, the secure energy outage event is defined as the event that the remaining energy used for UAVs’ hovering with the transmission time $T(X_{k},Y_{k})$ is larger than the total energy threshold, i.e., $p_{R_{k}}\;-\;P_{R_{k}}^{EH}=0$ (the transmit power at the *k*-th UAV equal to the harvested energy). Mathematically, this event can be described as

$$𝒪:P_{hover}T(X_{k},Y_{k})\geq E_{stor}+E_{marg}.$$ (15)

From the above formulation, it is clear that the operation of UAVs is not outage if and only if $P_{hover}T(X_{k},Y_{k})\geq E_{stor}$. Based on this fact, we aim to maximize the transmission time of communication by selecting one best UAV so that $P_{hover}T(X_{k},Y_{k})\;-\;E_{stor}$ is minimized. Since $E_{stor}$ is typically fixed according to the capacity size of the battery. Thus, the result above can be equivalently written as

$$\min_{k=\overset{―}{1,K}}\{P_{hover}T(X_{k},Y_{k})-E_{stor}\}=\frac{P_{hover}N/W}{\max_{k=\overset{―}{1,K}}\left\{C(X_{k},Y_{k})\right\}}-E_{stor}.$$ (16)

To maximize $C(X_{k},Y_{k})$, we introduce the following strategies:

1. The first strategy is to select the best UAV such that its end-to-end SNR from source to ground user is maximized, i.e.,
  $$k_{1}^{⋆}=\mathrm{\backslash arg}\max_{k=\overset{―}{1,K}}X_{k}.$$ (17)
2. The second strategy is to select the best UAV such that its SNR to the eavesdropper is minimized, i.e.,
  $$k_{2}^{⋆}=\mathrm{\backslash arg}\min_{k=\overset{―}{1,K}}Y_{k}.$$ (18)

## 3 Secure energy outage mathematical framework

This section analyzes the SEP of the considered system with three proposed UAV selection strategies. By definition, the SEP can be mathematically expressed as


$$\text{SEP}[k^{⋆}]=\mathrm{Pr}\left(P_{hover}T(X_{k^{⋆}},Y_{k^{⋆}})-E_{stor}\geq E_{marg}\right)$$

$$=\mathrm{Pr}\left(C(X_{k^{⋆}},Y_{k^{⋆}})\leq \frac{P_{hover}N}{W(E_{stor}+E_{marg})}≜\zeta \right),$$ (19)

where $k^{⋆}\in \{k_{i}^{⋆},i=1,2,3\}$. To facilitate finding $\text{SEP}[k^{⋆}]$, we introduce the following lemmas.

**Lemma 1.** Given $X_{k}=\min\{\gamma_{SR_{k}},\gamma_{R_{k}U}\}$, the CDF of *X*_*k*_ can be derived as

$$F_{X_{k}}(x)≃1-\frac{2}{\Gamma (m_{SR})}\sum_{m=0}^{m_{RU}-1}\frac{1}{m!}{\left(\frac{m_{RU}m_{SR}x}{\Omega_{RU}\Omega_{SR}\mu \epsilon \overset{―}{\gamma }}\right)}^{\frac{m+m_{SR}}{2}}{𝒦}_{m_{SR}-m}\left(2\sqrt{\frac{m_{RU}m_{SR}x}{\Omega_{RU}\Omega_{SR}\mu \epsilon \overset{―}{\gamma }}}\right).$$ (20)

*Proof:* Invoking the SNRs in (8) and (10) and $p_{R_{k}}=P_{R_{k}}^{EH}$, the CDF of *X*_*k*_ can be derived as


$$F_{X_{k}}(x)=\mathrm{Pr}\left(\min\{\gamma_{SR_{k}},\gamma_{R_{k}U}\}<x\right)=1-\mathrm{Pr}\left(\min\{\gamma_{SR_{k}},\gamma_{R_{k}U}\}>x\right)]$$

$$=1-\mathrm{Pr}\left((1-\epsilon )\overset{―}{\gamma }|h_{SR_{k}}{|}^{2}>x,\mu \epsilon \overset{―}{\gamma }|h_{SR_{k}}{|}^{2}|h_{R_{k}U}{|}^{2}>x\right)$$

$$=1-\mathrm{Pr}\left(|h_{SR_{k}}{|}^{2}>\frac{x}{(1-\epsilon )\overset{―}{\gamma }},|h_{R_{k}U}{|}^{2}>\frac{x}{\mu \epsilon \overset{―}{\gamma }|h_{SR_{k}}{|}^{2}}\right)$$

$$=1-\int_{\frac{x}{(1-\epsilon )\overset{―}{\gamma }}}^{\infty }\left[1-F_{|h_{R_{k}U}{|}^{2}}\left(\frac{x}{\mu \epsilon \overset{―}{\gamma }y}\right)\right]f_{|h_{SR_{k}}{|}^{2}}(y)dy.$$ (21)

By approximating $\frac{x}{(1-\epsilon )\overset{―}{\gamma }}≃0$, the CDF of *X*_*k*_ can be rewritten as

$$F_{X_{k}}(x)≃1-\sum_{m=0}^{m_{RU}-1}\frac{1}{m!}{\left(\frac{m_{RU}x}{\Omega_{RU}\mu \epsilon \overset{―}{\gamma }}\right)}^{m}\int_{0}^{\infty }y^{-m}\exp\left(-\frac{m_{RU}x}{\Omega_{RU}\mu \epsilon \overset{―}{\gamma }y}\right)\;\;\times \frac{y^{m_{SR}-1}}{\Gamma (m_{SR})}{\left(\frac{m_{SR}}{\Omega_{SR}}\right)}^{m_{SR}}\exp\left(-\frac{m_{SR}}{\Omega_{SR}}y\right)dy$$ (22)

$$=1-\sum_{m=0}^{m_{RU}-1}\frac{1}{m!}{\left(\frac{m_{RU}x}{\Omega_{RU}\mu \epsilon \overset{―}{\gamma }}\right)}^{m}\frac{1}{\Gamma (m_{SR})}{\left(\frac{m_{SR}}{\Omega_{SR}}\right)}^{m_{SR}}\;\;\times \int_{0}^{\infty }y^{m_{SR}-m-1}\exp\left(-\frac{m_{RU}x}{\Omega_{RU}\mu \epsilon \overset{―}{\gamma }y}-\frac{m_{SR}}{\Omega_{SR}}y\right)dy.$$ (23)

By applying [40, Eq. 3.471.9], one can get the solution for the integral above. ◻

**Lemma 2.** Given $Y_{k}=\gamma_{R_{k}E}$, the PDF and CDF of *Y* can be respectively derived as

$$f_{Y_{k}}(y)=\frac{2y^{-1}}{\Gamma (m_{SR})\Gamma (m_{RE})}{\left(\frac{m_{RE}m_{SR}y}{\Omega_{RE}\Omega_{SR}\mu \epsilon \overset{―}{\gamma }}\right)}^{\frac{m_{RE}+m_{SR}}{2}}{𝒦}_{m_{SR}-m_{RE}}\left(2\sqrt{\frac{m_{RE}m_{SR}y}{\Omega_{RE}\Omega_{SR}\mu \epsilon \overset{―}{\gamma }}}\right),$$ (24)

$$F_{Y_{k}}(y)=1-\frac{2}{\Gamma (m_{SR})}\sum_{m=0}^{m_{RE}-1}\frac{1}{m!}{\left(\frac{m_{RE}m_{SR}x}{\Omega_{RU}\Omega_{SR}\mu \epsilon \overset{―}{\gamma }}\right)}^{\frac{m+m_{SR}}{2}}{𝒦}_{m_{SR}-m}\left(2\sqrt{\frac{m_{RE}m_{SR}x}{\Omega_{RE}\Omega_{SR}\mu \epsilon \overset{―}{\gamma }}}\right).$$ (25)

*Proof:* Let $Q=|h_{SR_{k}}{|}^{2}|h_{R_{k}E}{|}^{2}$, the PDF of *Q* can be measured as


$$f_{Q}(q)=\int_{0}^{\infty }f_{|h_{SR_{k}}{|}^{2}}(x)f_{|h_{R_{k}E}{|}^{2}}(q/x)\frac{1}{x}dx$$

$$=\int_{0}^{\infty }\frac{x^{m_{SR}-1}}{\Gamma (m_{SR})}{\left(\frac{m_{SR}}{\Omega_{SR}}\right)}^{m_{SR}}\frac{(q/x{)}^{m_{RE}-1}}{\Gamma (m_{RE})}{\left(\frac{m_{RE}}{\Omega_{RE}}\right)}^{m_{RE}}\exp\left(-\frac{m_{RE}q}{\Omega_{RE}x}-\frac{m_{SR}}{\Omega_{SR}}x\right)\frac{dx}{x}$$

$$=\frac{q^{m_{RE}-1}{\left(\frac{m_{SR}}{\Omega_{SR}}\right)}^{m_{SR}}}{\Gamma (m_{SR})\Gamma (m_{RE})}{\left(\frac{m_{RE}}{\Omega_{RE}}\right)}^{m_{RE}}\int_{0}^{\infty }x^{m_{SR}-m_{RE}-1}\exp\left(-\frac{m_{RE}q}{\Omega_{RE}}\frac{1}{x}-\frac{m_{SR}}{\Omega_{SR}}x\right)dx$$

$$=\frac{2q^{-1}}{\Gamma (m_{SR})\Gamma (m_{RE})}{\left(\frac{m_{RE}m_{SR}}{\Omega_{RE}\Omega_{SR}}q\right)}^{\frac{m_{RE}+m_{SR}}{2}}{𝒦}_{m_{SR}-m_{RE}}\left(2\sqrt{\frac{m_{RE}m_{SR}}{\Omega_{RE}\Omega_{SR}}q}\right).$$ (26)

Invoking the SNRs in (8) and (10) while letting $p_{R_{k}}=P_{R_{k}}^{EH}$, one has that $Y_{k}=\overset{―}{\gamma }\mu \epsilon Q$. Thus, the PDF of *Y*_*k*_ can be derived as

$$f_{Y_{k}}(y)=\frac{1}{\overset{―}{\gamma }\mu \epsilon }f_{Q}\left(\frac{y}{\overset{―}{\gamma }\mu \epsilon }\right).$$ (27)

◻

**Lemma 3.** Given $I=\int_{0}^{\infty }f(x)dx$, its approximation can be expressed in terms of finite sum as

$$I≃\frac{\pi }{2L}\sum_{l=1}^{L}\frac{\sqrt{1-\phi_{l}^{2}}}{(1-\tau_{l}{)}^{2}}f\left(\frac{\tau_{l}}{1-\tau_{l}}\right),$$ (28)

where *L* is the accuracy trade-off parameter, $\tau_{l}=(\phi_{l}+1)/2$, and $\phi_{l}=\cos((2l-1)\pi /\left[2L\right])$.

*Proof:* This proof is proved by making the variable transform x=t/(1−t) to get $I=\int_{0}^{1}f(t/(1-t))/(1-t){\;}^{2}dt$ and then applying the Gauss-Chebyshev Quadrature method [41].

### 3.1 SEP with the first strategy

From (19) and (17), the SEP with the first strategy can be derived as


$$\text{SEP}[k_{1}^{⋆}]=\mathrm{Pr}\left(\frac{1+X_{k_{1}^{⋆}}}{1+Y_{k_{1}^{⋆}}}\leq \psi \right)$$

$$=\mathrm{Pr}\left(\frac{1+\max_{k=\overset{―}{1,K}}\{X_{k}\}}{1+Y_{\pi (k)}}\leq \psi \right),$$ (29)

where π(k) represents the random permutation of UAV selections and $\psi =2^{2\zeta }$.

**Proposition 1.** The analytical expression for the SEP with this strategy can be approximated as

$$\text{SEP}[k_{1}^{⋆}]≃\int_{0}^{\infty }{\left[F_{X_{k}}\left(\psi (1+y)-1\right)\right]}^{K}f_{Y_{k}}(y)dy$$ (30)

$$\approx \frac{\pi }{2L}\sum_{l=1}^{L}\frac{\sqrt{1-\phi_{l}^{2}}}{(1-\tau_{l}{)}^{2}}{\left[F_{X_{k}}\left(\frac{\psi }{1-\tau_{l}}-1\right)\right]}^{K}f_{Y_{k}}\left(\frac{\tau_{l}}{1-\tau_{l}}\right).$$ (31)

*Proof:* We begin the proof by rewriting the term $\text{SEP}[k_{1}^{⋆}]$ in (29) as


$$\text{SEP}[k_{1}^{⋆}]=\mathrm{Pr}\left(\max_{k=\overset{―}{1,K}}\{X_{k}\}\leq \psi (1+Y_{\pi (k)})-1\right)$$

$$=\int_{0}^{\infty }\mathrm{Pr}\left(\max_{k=\overset{―}{1,K}}\{X_{k}\}\leq \psi (1+y)-1\right)f_{Y_{\pi (k)}}(y)dy.$$ (32)

Using the connection $\mathrm{Pr}\left(\max_{k=\overset{―}{1,K}}\{X_{k}\}<x\right)=\mathrm{Pr}(X_{1}<x,...,X_{k}<x,...,X_{K}<x)=\prod_{k=1}^{K}\mathrm{Pr}(X_{k}<x)$, $F_{X}(x)=\mathrm{Pr}(X<x)$, and Lemma 3 yields the final SEP solution.

At high SNR, i.e., $\overset{―}{\gamma }\to \infty$, using the fact $\overset{―}{\gamma }+1≃\overset{―}{\gamma }$ can approximate the SEP in (29) as


$$\text{SEP}[k_{1}^{⋆}]≃\mathrm{Pr}\left(\frac{X_{k_{1}^{⋆}}}{Y_{k_{1}^{⋆}}}\leq \psi \right)=\mathrm{Pr}\left(\max_{k=\overset{―}{1,K}}\{X_{k}\}\leq \psi Y_{\pi (k)}\right)$$

$$=\mathrm{Pr}\left(\max_{k=\overset{―}{1,K}}\left\{|h_{SR_{k}}{|}^{2}\min\{(1-\epsilon ),\mu \epsilon |h_{R_{k}U}{|}^{2}\}\right\}\leq \psi \mu \epsilon |h_{SR_{\pi (k)}}{|}^{2}|h_{R_{\pi (k)}E}{|}^{2}\right)$$

$$=\int_{0}^{\infty }{\left[F_{U_{k}}\left(\psi \mu \epsilon v\right)\right]}^{K}f_{V_{k}}(v)dv,$$ (33)

where $U_{k}≜|h_{SR_{k}}{|}^{2}\min\{(1-\epsilon ),\mu \epsilon |h_{R_{k}U}{|}^{2}\}$, $V_{k}≜|h_{SR_{\pi (k)}}{|}^{2}|h_{R_{\pi (k)}E}{|}^{2}$, and their CDF and PDF of *U*_*k*_ and $V_{k}$ can be derived similar to that of *X*_*k*_ and *Y*_*k*_ as

$$F_{U_{k}}(u)≃1-\frac{2}{\Gamma (m_{SR})}\sum_{m=0}^{m_{RU}-1}\frac{1}{m!}{\left(\frac{m_{RU}m_{SR}u}{\Omega_{RU}\Omega_{SR}\mu \epsilon }\right)}^{\frac{m+m_{SR}}{2}}{𝒦}_{m_{SR}-m}\left(2\sqrt{\frac{m_{RU}m_{SR}u}{\Omega_{RU}\Omega_{SR}\mu \epsilon }}\right),$$ (34)

$$F_{V_{k}}(v)=\frac{2v^{-1}}{\Gamma (m_{SR})\Gamma (m_{RE})}{\left(\frac{m_{RE}m_{SR}v}{\Omega_{RE}\Omega_{SR}}\right)}^{\frac{m_{RE}+m_{SR}}{2}}{𝒦}_{m_{SR}-m_{RE}}\left(2\sqrt{\frac{m_{RE}m_{SR}v}{\Omega_{RE}\Omega_{SR}}}\right).$$ (35)

Making use of Lemma 3, the asymptotic SEP with the first strategy can be derived as

$$\text{SEP}[k_{1}^{⋆}]\approx \frac{\pi }{2L}\sum_{l=1}^{L}\frac{\sqrt{1-\phi_{l}^{2}}}{(1-\tau_{l}{)}^{2}}{\left[F_{U_{k}}\left(\frac{\psi \mu \epsilon \tau_{l}}{1-\tau_{l}}\right)\right]}^{K}f_{V_{k}}\left(\frac{\tau_{l}}{1-\tau_{l}}\right).$$ (36)

**Remark 1.** Comparing the results in (36) with that of (31) shows that the SEP is independent with $\overset{―}{\gamma }$, showing that the SEP does not improve with an increase in the transmit SNR.

### 3.2 SEP with the second strategy

From (19) and (18), the SEP with the second strategy can be derived as

$$\text{SEP}[k_{2}^{⋆}]=\mathrm{Pr}\left(\frac{1+X_{k_{2}^{⋆}}}{1+Y_{k_{2}^{⋆}}}\leq \psi \right)=\mathrm{Pr}\left(X_{\pi (k)}\leq \psi (1+\min_{k=\overset{―}{1,K}}Y_{k})-1\right).$$ (37)

**Proposition 2.** The analytical expression for the SEP with this strategy can be approximated as

$$\text{SEP}[k_{2}^{⋆}]=\int_{0}^{\infty }F_{X_{k}}\left(\psi (1+z)-1\right)Kf_{Y_{k}}(z)[1-F_{Y_{k}}(z){]}^{K-1}dz$$ (38)

$$≃\frac{K\pi }{2L}\sum_{l=1}^{L}\frac{\sqrt{1-\phi_{l}^{2}}}{(1-\tau_{l}{)}^{2}}F_{X_{k}}\left(\frac{\psi }{1-\tau_{l}}-1\right)f_{Y_{k}}\left(\frac{\tau_{l}}{1-\tau_{l}}\right){\left[1-F_{Y_{k}}\left(\frac{\tau_{l}}{1-\tau_{l}}\right)\right]}^{K-1}.$$ (39)

*Proof:* Let $Z=\min_{k=\overset{―}{1,K}}Y_{k}$, the CDF of *Z* can be derived as


$$F_{Z}(z)=\mathrm{Pr}(Z<z)=1-\mathrm{Pr}(Z>z)$$

$$=1-\prod_{k=1}^{K}\mathrm{Pr}(Y_{k}>z)=1-[1-F_{Y_{k}}(z){]}^{K}.$$ (40)

Based on this, the PDF of *Z* can be derived as [42]


$$f_{Z}(z)=\frac{\partial }{\partial z}F_{Z}(z)$$

$$=\sum_{k=1}^{K}f_{Y_{k}}(z)\prod_{l=1,l\neq k}^{K}[1-F_{Y_{l}}(z)]=Kf_{Y_{k}}(z)[1-F_{Y_{l}}(z){]}^{K-1}.$$ (41)

Following that, from (37) and Lemma 3, one can get the final solution for the SEP.

At high SNR, i.e., $\overset{―}{\gamma }\to \infty$, using the fact $\overset{―}{\gamma }+1≃\overset{―}{\gamma }$ can approximate the SEP in (29) as


$$\text{SEP}[k_{2}^{⋆}]≃\mathrm{Pr}\left(\frac{X_{k_{2}^{⋆}}}{Y_{k_{2}^{⋆}}}\leq \psi \right)=\mathrm{Pr}\left(X_{\pi (k)}\leq \psi \min_{k=\overset{―}{1,K}}\{Y_{k}\}\right)$$

$$=\mathrm{Pr}\left(\left\{|h_{SR_{\pi (k)}}{|}^{2}\min\{(1-\epsilon ),\mu \epsilon |h_{R_{\pi (k)}U}{|}^{2}\}\right\}\leq \psi \mu \epsilon \min_{k=\overset{―}{1,K}}\{|h_{SR_{k}}{|}^{2}|h_{R_{k}E}{|}^{2}\}\right)$$

$$=\int_{0}^{\infty }F_{U_{k}}\left(\psi \mu \epsilon v\right)Kf_{V_{k}}(v)[1-F_{V_{l}}(v){]}^{K-1}dv$$

$$\approx \frac{\pi K}{2L}\sum_{l=1}^{L}\frac{\sqrt{1-\phi_{l}^{2}}}{(1-\tau_{l}{)}^{2}}F_{U_{k}}\left(\frac{\psi \mu \epsilon \tau_{l}}{1-\tau_{l}}\right)f_{V_{k}}\left(\frac{\tau_{l}}{1-\tau_{l}}\right){\left[1-F_{V_{l}}\left(\frac{\tau_{l}}{1-\tau_{l}}\right)\right]}^{K-1},$$ (42)

which is approximated using Lemma 3.

**Remark 2.** By comparing the results in (42) with that of (39), we found that the SEP with this strategy is also independent with $\overset{―}{\gamma }$.

## 4 Numerical results and discussions

  This section provides some results using the Monte-Carlo simulation to verify the prior mathematical framework. The technological advancement of our code lies in the implementation of symbolic calculations in Matlab, which helps achieve high-precision results without simulation evaluation. Without loss of generality, we list all used parameters in Table 3.

**Table 3:** Simulation Parameters [39].

Notation	Description	Value
*w*	UAV weights (frame 1.5 kg, battery and payload 2 kg)	3.5 kg
δ	the number of rotors	4
$E_{stor}$	Energy storage (4-cell 14.8 V/10 Ah lithium polymer battery)	532800 J
$E_{marg}$	Marginal energy budget (10% of Energy storage)	53280 J
$G_{AB}$	Transceiver antenna gain	$G_{SR}=6$ dB $G_{RE}=G_{RU}=4$ dB
*f*	Frequency usage	1 GHz
*B*	Bandwidth	10 MHz
*N*	Message size	50 MHz
$\sigma^{2}$	Noise variance $\sigma^{2}=-174+10\log_{10}(B)$	–94 dB
ϵ	Power splitting coefficient	0.7
μ	Energy conversion efficiency	0.7
ρ	Pathloss coefficient	3
$m_{AB}$	Fading parameters	$m_{SR}=m_{RU}=2$ $m_{RE}=1$
$d_{AB}$	Distance parameters	$d_{SR}=d_{RU}=20$ $d_{RE}=15$
*L*	Trade-off parameter	200

**Fig 2:**
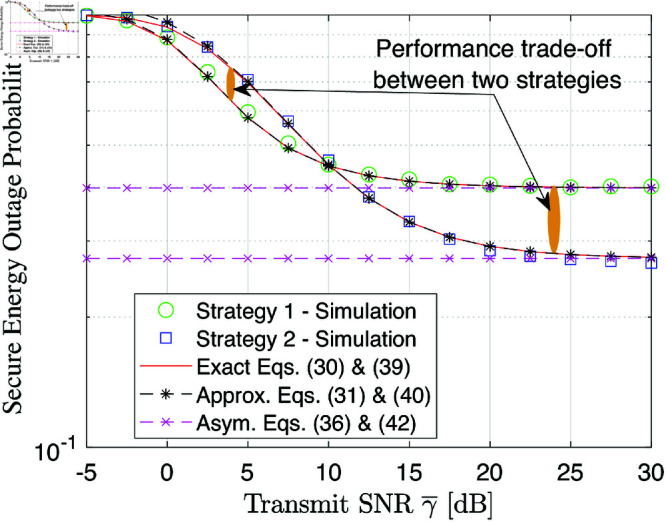
SEP performance versus the transmit SNR $\overset{―}{\gamma }$ [dB].

Fig 2 illustrates the SEP performance of the considered system as a function of the transmit SNR $\overset{―}{\gamma }$. As observed, the analytical outcomes produced by (30) and (38) as well as the approximated ones show a great agreement with the simulation results (denoted by blue and green markers). Also, the SEP decreases as the value of $\overset{―}{\gamma }$ increases from -5 dB to 20 dB and then becomes saturation. This trend can be explained by a larger value of $\overset{―}{\gamma }$ resulting in better secure signal reception, as depicted in (11), and when $\overset{―}{\gamma }$ becomes large enough, $C(X_{k},Y_{k})\approx {\left[0.5\log_{2}\left(X_{k}/Y_{k}\right)\right]}^{+}$, which is a constant value. This observation highlights that the SEP performance is only improved with the small and moderate transmit SNRs. On the other hand, it is also observed that employing strategy 1 achieves better SEP performance than strategy 2 at low and moderate SNR but this trend is reversed with the other SNR region.

**Fig 3:**
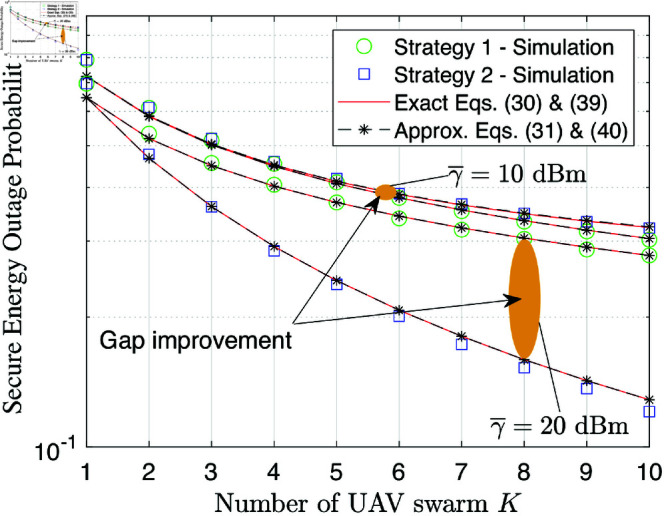
SEP performance versus the number of UAV swarms K at $\overset{―}{\gamma }=10$ [dB].

Fig 3 depicts the SEP performance as a function of the number of UAV swarms *K*. It is observed that an increase in *K* leads to a decrease in the SEP. This is because higher *K* values result in higher UAV selection ability with fewer energy outage events, which significantly improves the hovering activity. In addition, it is clear that under configurations of small SNR, i.e., $\overset{―}{\gamma }=10$ dB, the performance gap between strategies 1 and 2 is minor. Meanwhile, the performance gap between strategies 1 and 2 with the large SNR $\overset{―}{\gamma }=20$ dB becomes significant when *K* increases. This observation highlights that deploying a larger number of UAV swarms makes sense if and only if strategy 2 is used.

**Fig 4:**
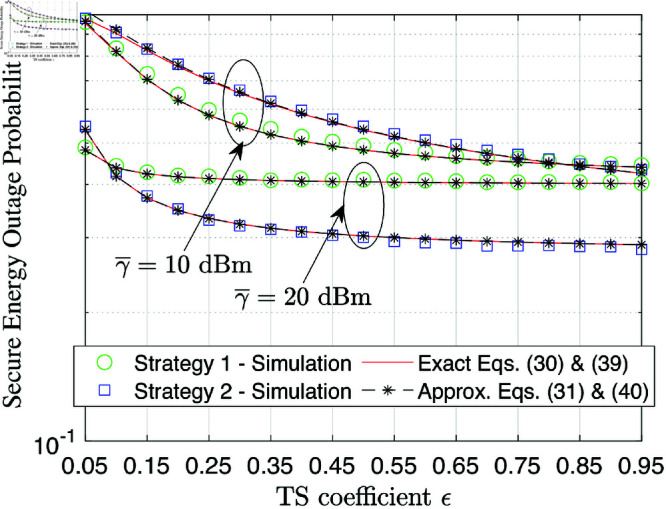
SEP performance versus the PA coefficient ϵ at $\overset{―}{\gamma }=10$.

Fig 4 illustrates the SEP performance as a function of the PA coefficient ϵ. It can be seen that when the transmit SNR is small, e.g., $\overset{―}{\gamma }=10$ dB, the variation of ϵ from 0.05 to 0.95 helps improve the SEP. However, when the transmit SNR is large, e.g., $\overset{―}{\gamma }=20$ dB, the SEP is only improved with the variation of ϵ from 0.05 to 0.35 and keeps stable with the other range of ϵ. This means that allocating more power to energy harvesting at the UAV is only beneficial at small transmission SNR but not at high SNR regimes.

**Fig 5:**
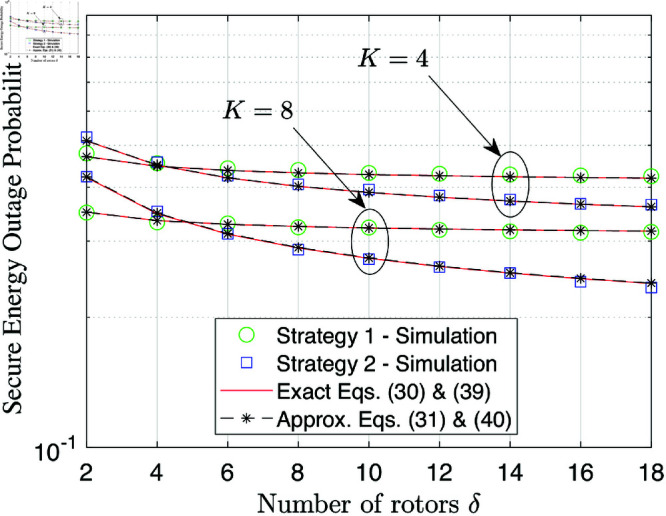
SEP performance versus the number of rotors of UAVs δ at $\overset{―}{\gamma }=10$ dB.

Fig 5 depicts the SEP performance as a function of the number of UAV rotors, δ. As observed, increasing δ reduces the SEP in strategy 2, primarily due to the decrease in hovering power consumption $P_{hover}$, as described in (13). This reduction allows more harvested energy to be allocated to secure communication, improving overall system performance. In contrast, the SEP for strategy 1 remains largely unaffected by δ, suggesting that its performance is more influenced by factors such as UAV selection and channel conditions rather than power efficiency improvements from additional rotors. This highlights that while rotor optimization can enhance security in certain scenarios, its impact varies depending on the chosen UAV selection strategy.

**Fig 6:**
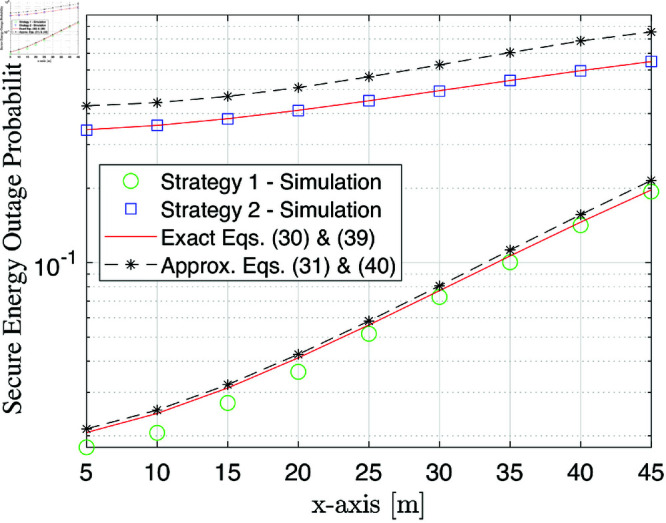
SEP performance versus the movement of UAV according to $x_{R}$ [m] at $\overset{―}{\gamma }=20$ dB.

Next, to assess the impact of UAV location, we consider a scenario where the source, selected UAV, ground user, and eavesdropper are positioned at $S(0,0,0),R(x_{R},0,z_{R}),U(0,50,0)$, and E(30,0,0). This setting leads to $d_{SR}=\sqrt{x_{R}^{2}+z_{R}^{2}}$, $d_{RU}=\sqrt{x_{R}^{2}+50^{2}+z_{R}^{2}}$, and $d_{RE}=\sqrt{(x_{R}-30){\;}^{2}+z_{R}^{2}}$. Based on this, we proceed with the evaluation of the SEP under different hovering trajectories of the selected UAV. Specifically, Fig 6 illustrates the SEP performance as a function of the movement of UAV according to x-axis $x_{R}$ when $z_{R}=50$ m. From this figure, we can observe that when a UAV moves close to the ground user, the SEP curves tend to increase. This is because the communication link between the source and the UAV is reduced and thus the amount of energy harvested is also reduced, increasing the transmission power used for the cooperative communication phase. That is to say, the secure achievable rate $C(X_{k},Y_{k})$ decreases or the time frame *T* for communication increases, increasing the SEP.

These results highlight the trade-off between UAV positioning and security performance. While proximity to the ground user may improve direct communication, it negatively impacts energy availability and overall secrecy. Thus, careful trajectory optimization is essential to balance energy efficiency and secure communication.

**Fig 7:**
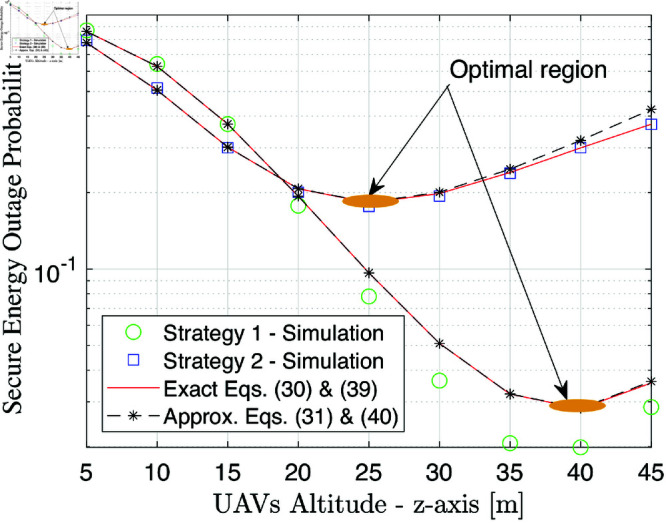
SEP performance versus the movement of UAV according to $z_{R}$ [m] at $\overset{―}{\gamma }=20$ dB.

Fig 7 illustrates the SEP performance as a function of the movement of UAV according to z-axis $z_{R}$ when $x_{R}=25$ m. As observed, the SEP curves tend to decrease with $z_{R}$ varying from 5 to 25 for strategy 2 and from 5 to 40 for strategy 1 before increasing with the other range of $z_{R}$. This trend indicates the existence of an optimal altitude where SEP is minimized.

The improvement in SEP at lower altitudes can be attributed to the enhanced source-UAV link quality, resulting in better energy harvesting and stronger cooperative communication. However, as altitude increases beyond a certain point, the increased path loss and weaker signal reception degrade secrecy performance, leading to a rise in SEP. These results underscore the importance of altitude optimization in UAV-assisted secure communication. Since our mathematical framework can be expressed as a unique function of location, it is possible to reach this optimal solution by exploiting simple search methods without computational complexity like bisection or golden search.

## 5 Conclusion

This paper provided an effective approach to evaluate the SEP performance of UAV swarm-aided energy harvesting networks operating with the SWIPT-PS mechanism. Under two proposed UAV selection schemes, we analyzed the SEP performance by deriving the approximate and asymptotic expressions. Our results demonstrate that the number of UAV swarms has a clear effect on the SEP performance; particularly, the greater the number of UAVs, the better the system’s performance. Besides, we also varied the power-splitting coefficient to examine its effect on system performance and the results indicate that increasing this coefficient only improves SEP performance with lower transmit SNR values. Moreover, we quantified the impact of UAV trajectories, where the SEP is improved when the UAV flies closer to the source and becomes minimal if its altitude that optimized.

In this work, we have analyzed the performance of SEP in UAV-assisted energy harvesting networks. However, our investigation is currently limited to single-input single-output communication scenarios, leaving issues related to multi-antenna transceivers and hardware impairments unaddressed. Additionally, practical eavesdropping scenarios can range from non-colluding to colluding, making it worthwhile to explore these cases further. Furthermore, interference from co-channel signals may significantly impact the reception quality at both the UAV and the destination, potentially affecting the performance of energy outage events. Therefore, addressing these challenges presents an exciting direction for future research.Supporting information

## Supporting information

S1 File The simulation code used in this work can be found at: MatlabCode. (ZIP)
